# Esculetin Ameliorates Carbon Tetrachloride-Mediated Hepatic Apoptosis in Rats

**DOI:** 10.3390/ijms12064053

**Published:** 2011-06-16

**Authors:** Yun-Chen Tien, Jung-Chun Liao, Chuan-Sung Chiu, Tai-Hung Huang, Chih-Yang Huang, Wen-Te Chang, Wen-Huang Peng

**Affiliations:** 1 School of Chinese Pharmaceutical Sciences and Chinese Medicine Resources, College of Pharmacy, China Medical University, 91 Hsueh-Shih road, Taichung 404, Taiwan; E-Mails: austion23@yahoo.com.tw (Y.-C.T.); chu4224610@yahoo.com.tw (C.-S.C.); wtchang@mail.cmu.edu.tw (W.-T.C.); 2 School of Pharmacy, College of Pharmacy, China Medical University, 91 Hsueh-Shih road, Taichung, Taichung 404, Taiwan; E-Mails: jcliao@mail.cmu.edu.tw (J.-C.L.); hthung@mail.cmu.edu.tw (T.-H.H.); 3 Hsin Sheng College of Medical Care and Management, Taoyuan, 325, Taiwan; 4 Graduate Institute of Basic Medical Science, China Medical University, 91, Hsueh-Shih road, Taichung 404, Taiwan; E-Mail: cyhuang@mail.cmu.edu.tw

**Keywords:** esculetin, carbon tetrachloride, apoptosis

## Abstract

Esculetin (ESC) is a coumarin that is present in several plants such as *Fraxinus rhynchophylla* and *Artemisia capillaris*. Our previous study found that *FR* ethanol extract (FR_EtOH_) significantly ameliorated rats’ liver function. This study was intended to investigate the protective mechanism of ESC in hepatic apoptosis in rats induced by carbon tetrachloride. Rat hepatic apoptosis was induced by oral administration of CCl_4_. All rats were administered orally with CCl_4_ (20%, 0.5 mL/rat) twice a week for 8 weeks. Rats in the ESC groups were treated daily with ESC, and silymarin group were treated daily with silymarin. Serum alanine aminotransferase (ALT), aspartate aminotransferase (AST) as well as the activities of the anti-oxidative enzymes glutathione peroxidase (GPx), superoxide dismutase (SOD), and catalase in the liver were measured. In addition, expression of liver apoptosis proteins and anti-apoptotic proteins were detected. ESC (100, 500 mg/kg) significantly reduced the elevated activities of serum ALT and AST caused by CCl_4_ and significantly increased the activities of catalase, GPx and SOD. Furthermore, ESC (100, 500 mg/kg) significantly decreased the levels of the proapoptotic proteins (t-Bid, Bak and Bad) and significantly increased the levels of the anti-apoptotic proteins (Bcl-2 and Bcl-xL). ESC inhibited the release of cytochrome c from mitochondria. In addition, the levels of activated caspase-9 and activated caspase-3 were significantly decreased in rats treated with ESC than those in rats treated with CCl_4_ alone. ESC significantly reduced CCl_4_-induced hepatic apoptosis in rats.

## 1. Introduction

Although many chronic viral hepatitis patients have been treated with interferon, the results of the therapy have not always been satisfactory. Chronic viral hepatitis (especially type C) in Taiwan [1], as well as in the whole of Asia [2], ends with the development of hepatocellular carcinoma, usually in a fibrotic or cirrhotic liver. ESC (6,7-dihydroxy-2H-1-benzopyran-2-one; ESC), one of the potent coumarin-derived antioxidants, reduces oxidative stress in various modes such as reduction of neutrophil-dependent superoxide anion generation [3], free radical scavenging [4,5] and reduction of lipid peroxidation [6]. ESC has been shown to have an anti-inflammatory effects *in vivo* [7] and anti-proliferative effects on vascular smooth muscle cells [8]. Furthermore, ESC has been reported to inhibit oxidative damage induced by *tert*-butyl hydroperoxide in rat liver [9]. It has been isolated from various plants such as *Artemisia capillaries*, *Citru slimonia* and *Euphorbia lathyris.* In our preliminary study, ESC was demonstrated to inhibit CCl_4_-induced acute liver injury in rats. However, there is still little information on the effect of ESC on CCl_4_-induced fibrosis in rats.

Carbon tetrachloride (CCl_4_) is extensively used to induce lipid peroxidation and toxicity. CCl_4_ is metabolized by cytochrome P450 2E1 to the trichloromethyl radical (CCl3^−^), which is assumed to initiate free radical-mediated lipid peroxidation leading to the accumulation of lipid-derived oxidation products that cause liver injury [10]. Polyunsaturated fatty acids (PUFAs) in membrane lipids are especially susceptible to free radical-initiated peroxidation. PUFAs in phospholipids of the endothelium reticulum were decreased following CCl_4_ administration *in vivo* [11]. Liver fibrosis as well as the end-stage of liver fibrosis and cirrhosis represents the final common pathway of virtually all chronic liver diseases [12]. Its progression leads to cirrhosis and liver cancer [13].

A number of investigators have previously demonstrated that antioxidants prevent CCl_4_ toxicity, particularly hepatotoxicity, by inhibiting lipid peroxidation [14] and increasing the activities of antioxidant enzymes [15]. Silymarin is a group of flavones extracted from *Silubum marianum* L and is a strong anti-oxidant [16] and an effective agent for liver protection and liver cell regeneration.

Apoptosis is a genetically encoded form of cell suicide central to the development and homeostasis of multicellular organisms [17,18]. Researchers once assumed that the activation of endonucleases and specific proteases (such as caspases) reflect the key mechanism of apoptosis [19]. The mitochondrial pathway is partly dependent on the release of cytochrome c. After release from mitochondria to the cytosol, cytochrome c binds to apoptosis-activating factor-1 (Apaf-1), ATP (or dATP), and possibly a cytosolic protein (Apaf-3), and activates caspase 9, which in turn stimulates caspase 3 activity [20,21]. Bcl-2 proteins act on mitochondria to regulate apoptosis. The Bcl-2 family consists of both cell death promoters and preventers, including the anti-apoptotic proteins Bcl-2, Bcl- xL, Mcl-1, A1/Bfl-1 and Bcl-W, and the pro-apoptotic members Bax, Bak, Bad, Bik, Bid, Hrk and Bok [22]. Bcl-2 and Bcl-xL prevent cytochrome c from entering the cytosol, either by blocking release or binding to the cytochrome in a direct or indirect fashion, and consequently inhibiting activation of the downstream caspase cascade. Reactive oxygen species (ROS), which induce the onset of the mitochondrial permeability transition (MPT), play an important role in mitochondrial apoptosis. Activation of MPT is a major controlling mechanism in some apoptotic systems, and it also contributes to the release of cytochrome c and other apoptogenic proteins [20].

In the present study, we examined the effect of ESC on CCl_4_-induced liver apoptosis in rats. The hepatoprotective effect of ESC was judged by histological and biochemical values including the activities of serum aspartate amino transferase (sAST), serum alanine amino transferase (sALT), superoxide dismutase (SOD), glutathione peroxidase (GPx) and catalase in the liver. We also examined the effects of ESC on the regulation of the proteins in the Mitochondrial-Dependent Apoptotic Pathway in CCl_4_-induced liver apoptosis. In this study, silymarin was used as a positive control drug.

## 2. Results and Discussion

### 2.1. Serum ALT and AST Activities

**Figure 1:**
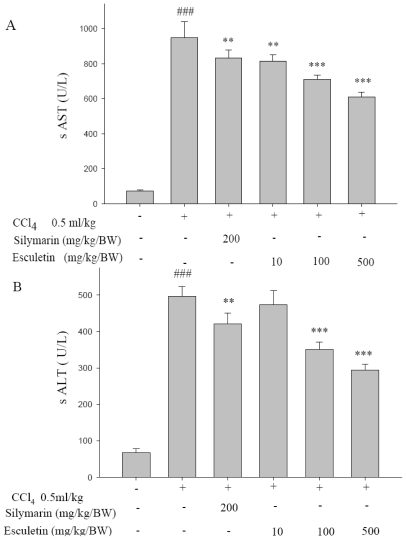
Effect of esculetin and silymarin on serum (**A**) alanine aminotransferase (ALT) and (**B**) aspartate aminotransferase (AST) levels in CCl_4_-treated rats. Data are expressed as mean ± SD (n = 10). *** *p* < 0.001, ** *p* < 0.01 as compared with the CCl_4_ group. ### *p* < 0.001 as compared with the Control group. Statistical analysis was carried out by one-way analysis of variance followed by Scheffe’s multiple range tests.

The serum activities of ALT and AST were significantly elevated in the CCl_4_-treated group (*p* < 0.001) where ESC (100 and 500 mg/kg BW) significantly decreased the activities of serum ALT and AST (*p* < 0.001). The effect of supplement of silymarin was similar to that observed for the ESC-treated group (*p* < 0.001) (Figure 1).

Serum levels of ALT and AST are the most commonly used biochemical markers of liver injury [23]. Hepatic damage such as liver fibrosis, cirrhosis and liver atrophy can initiate the actions of regeneration, and thus increase the weight of liver [24]. In this study, it is shown that chronic administration of CCl_4_ led to a marked increase in ALT and AST levels. In addition, when liver injury was evaluated by a histological approach [25], infiltration of mononuclear cells around the Glisson’s sheath after 8 weeks of CCl_4_ treatment was detected. Furthermore, CCl_4_ was also found to cause a variety of histological changes in the liver, including centrizonal necrosis, portal inflammation, and Kupffer cell hyperplasia. These histological changes as well as the increase in hepatic enzymes were significantly attenuated by ESC (100 and 500 mg/kg/BW) (Table 1).

### 2.2. Changes in Liver Histopathology

**Figure 2:**
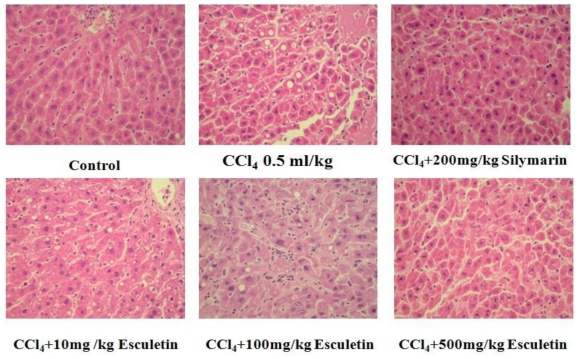
Photomicrographs of liver sections taken from rat with CCl_4_-induced liver damage after pretreatment with esculetin or silymarin for 8 weeks. Liver damage was characterized by fatty degeneration, cell necrosis and mitosis. (Hematoxylin/Eosin staining, 200×) by Knodell *et al.* [25].

As shown in Figure 2, normal hepatic architecture with a central vein and radiating hepatic cords were seen. In the CCl_4_-treated group, sections showed degeneration and necrosis, fibrosis, hepatocyte infiltration containing neutrophils and mononuclear cells. In addition, central-to-central bridging necrosis was also noted. Moreover, treatment with of ESC (100 and 500 mg/kg BW) or silymarin significantly decreased the degree of inflammation, necrosis and fibrosis in CCl_4_-treated groups rats.

### 2.3. Hepatic Catalase, SOD and GPx Activities

We next explored the effect of ESC on catalase, SOD and GPx activities in CCl_4_-induced hepatic fibrosis rats. It was found that the activities of SOD, GPx and catalase were markedly increased in rats treated with ESC (100 and 500 mg/kg BW). Hepatic GPx activities were significantly increased in rats treated with ESC (100 and 500 mg/kg BW) and silymarin (200 mg/kg BW) (*P* < 0.001) (Table 1).

Living tissue is a major defense mechanism involving anti-oxidative enzymes that convert active oxygen molecules into non-toxic compounds [26,27]. Anti-oxidative enzymes, such as SOD, GPx and catalase, are easily inactivated by lipid peroxides or reactive oxygen species during CCl_4_ exposure [28]. Glutathione (GSH) is a known radical scavenger, and GSH-related enzymes, such as GPx and GR, play detoxifying and anti-oxidative roles through conjugation with glutathione or free radical reduction [29]. There are three classes of enzymes known to provide protection against reactive oxygen species: SOD, catalase, and GPx [30]. We have demonstrated that ESC (100 and 500 mg/kg/BW) significantly reversed the CCl_4_-induced decrease in activities of anti-oxidative enzymes, such as Catalase, SOD and GPx. These results suggested that ESC reduced CCl_4_-induced hepatic fibrosis in rats, probably by exerting a protective effect against hepatocellular fibrosis with its free-radical scavenging ability.

### 2.4. Changes in Caspase-9, Caspase-3 and Cytochrome c Protein Levels in CCl_4_ -Treated Rats

**Figure 3:**
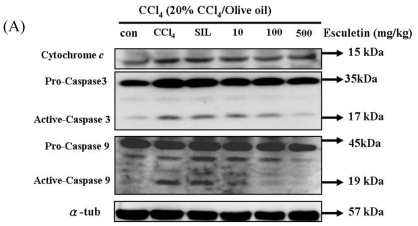

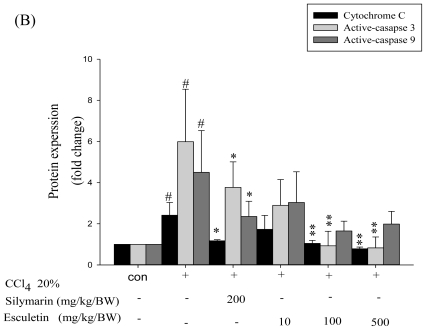
(**A**) CCl_4_ chronic treated rats (for 8 weeks) were harvested and lysed. Total protein of cell extracts was separated by 12% SDS-PAGE, transferred to PVDF membranes, and immunoblotted with antibodies against proteins as indicated. Equal loading was assessed with an anti-tubulin antibody. Proteins (pro-caspase-9, active-caspase-9 and pro-caspase-3, active-caspase-3, and cytochrome c) extracted from liver tissue were measured by Western blotting analysis. Equal loading was assessed with an anti-α-tubulin antibody. (**B**) The expression of cytochrome c, active-caspase-3 and active-caspase-9 proteins. * *p* < 0.05, ** *p* < 0.01 as compared with the CCl_4_ group; # *p* < 0.05 as compared with the Control group.

To further investigate the downstream signal components of cytochrome c release in the mitochondrial-dependent signaling pathway, pro-caspase-9 levels, activated caspase-9, pro-caspase-3 levels and activated caspase-3 were measured by Western blotting. Compared with the control group, it was found that the activated caspase-3, activated caspase-9, and cytochrome *c* levels were significantly increased in the CCl_4_-treated group. In addition, the activated-caspase-9 levels and activated-caspase-3 levels were significantly decreased in ESC-treated groups (100 and 500 mg/kg) (Figure 3).

Carbon tetrachloride is a common hepatotoxin used in liver injury research. Early studies showed that the damage induced by CCl_4_ in liver is partly involved in the apoptosis pathway *in vivo*. At least 2 different apoptosis pathways—the Mitochondrial pathway and the Death-receptor pathway—lead to caspase activation [31]. Although past reports have disclosed caspase 3 activation and other histopathological changes in CCl_4_-induced apoptotic hepatocytes [32,33], little is known about the precise molecular mechanisms of apoptosis induction. Cytochrome *c* release will form a complex with pro-caspase-9 and its cofactor Apaf-1 (apoptotic protease-activating factor-1). Therefore, it is responsible for activating caspase-9, which further activates caspase-3 and executes the apoptotic program.

### 2.5. Changes in Bcl-2 Family Components in CCl_4_-Treated Rats

**Figure 4:**
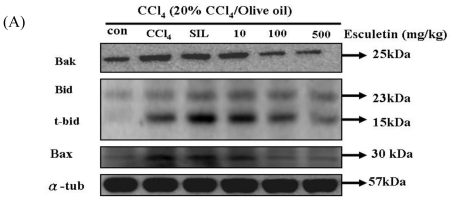

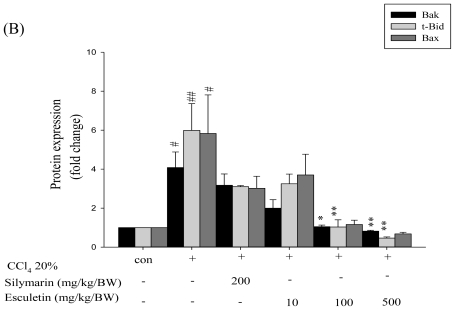
CCl_4_ chronic treated rats (for 8 weeks) were harvested and lysed. Total protein of cell extracts was separated by 12% SDS-PAGE, transferred to PVDF membranes, and immunoblotted with antibodies against proteins as indicated. Equal loading was assessed with an anti-α-tubulin antibody. (**A**) Proteins (Bad, t-Bid, and Bax) extracted from liver tissue were measured by Western blotting analysis. Equal loading was assessed with an anti-α-tubulin antibody; (**B**) The expression of Bak, Bax and t-Bid proteins. *p < 0.05, ** *p* < 0.01 as compared with the CCl_4_ group; #*p* < 0.05, ##*p* < 0.01 as compared with the Control group.

To further understand the effect of ESC on the mitochondrial-dependent apoptotic pathway, we examined the levels of three pro-apoptotic proteins (Bak, t-Bid, and Bax) in the liver tissue of the CCl_4_-treated group. Bak, t-Bid and Bax levels in the CCl4-treated group were significantly higher than those in the control group. Also, Bak, t-Bid and Bax levels were significantly decreased in the ESC-treated groups (100 and 500 mg/kg) (Figure 4).

**Figure 5:**
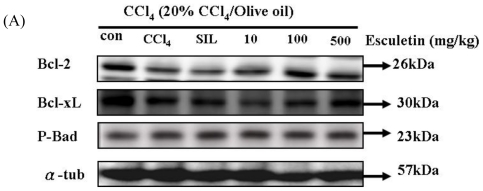

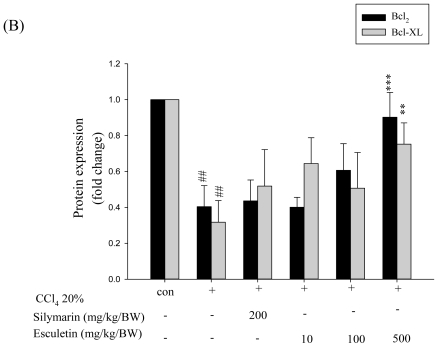
CCl_4_ chronic treated rats (for 8 weeks) were harvested and lysed. Total protein of cell extracts was separated by 12% SDS-PAGE, transferred to PVDF membranes, and immunoblotted with antibodies against proteins as indicated. Equal loading was assessed with an anti-tubulin antibody. **(A)** The protein products of anti-apoptotic Bcl-2, Bcl-x L, p-Bad. Proteins extracted from liver tissue were measured by Western blotting analysis. Equal loading was assessed with an anti-tubulin antibody; **(B)** The expression of Bcl-2 and Bcl- x L proteins. ** *p* < 0.01, *** *p* < 0.001as compared with the CCl_4_ group; # *p* < 0.05, ## *p* < 0.01 as compared with the Control group.

We examined the levels of three anti-apoptotic Bcl-2 proteins, namely Bcl-2, p-Bad and Bcl-xL, in liver tissue of apoptosis rats. Bcl-2 and Bcl-x L levels were significantly lower in the CCl_4_-treated group than those in the control group. Bcl-xL and Bcl-2, but not p-Bad, were significantly increased in ESC-treated groups (100 and 500 mg/kg) (Figure 5).

Mitochondria are known to be a vulnerable target of various toxins and oxidative stress. The mitochondrial apoptotic pathway is regulated by the Bcl-2 family of proteins, which consist of both anti-apoptotic (such as Bcl-2) and pro-apoptotic (such as Bax) proteins [34]. Activation of the pro-apoptotic Bcl-2 family of proteins such as Bax is known to play an important role in the oxidative stress-induced apoptotic pathway [35]. It has been shown that Bax can translocate from the cytosol to mitochondria and exhibit conformational change under the apoptotic process.

In this study, we have also demonstrated that ESC increased the levels of Bcl-2 and Bcl-x L and decreased the levels of Bak, Bax, t-Bid, cytochrome c, activated caspase-9, and activated caspase-3 proteins in rats exposed to CCl_4_. Our data show that Bax protein content markedly increased in rats receiving CCl_4_ alone, suggesting that oxidative stress caused by CCl_4_ administration has activated Bax, Bak and t-Bid. We also found that Bcl-2 protein content markedly increased in rats receiving CCl_4_ alone. Administration of ESC effectively decreased the levels of Bak, Bax, and t-Bid.

**Figure 6:**
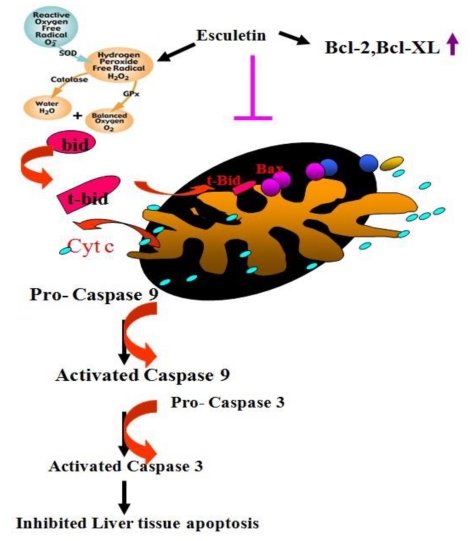
Proposed mechanisms of action of esculetin on liver apoptosis. Esculetin suppresses the mitochondrial-dependent apoptotic pathway by inhibiting the release of mitochondrial pro-apoptotic proteins Bax, Bak, and t-Bid which in turn inhibits the release of cytochrome c, caspase-9 and caspase 3, there by inducing the release of anti-apoptotic proteins Bcl-2 and Bcl-x L.

In summary, mitochondria-initiated apoptosis triggered by ROS plays an important role in CCl_4_-induced hepatotoxicity in rats. ESC significantly reduced CCl_4_-induced hepatic fibrosis in rats, probably by exerting a protective effect against hepatocellular fibrosis with its free-radical scavenging ability and inhibiting the Mitochondrial-Dependent Apoptotic Pathway in CCl_4_-induced liver apoptosis in rats (Figure 6).

## 3. Experimental Section

### 3.1. Animals

Male Sprague-Dawley rats were obtained from the BioLASCO Taiwan Co., Ltd., and fed with a standard laboratory diet and tap water *ad libitum*. The experimental animals were housed in an air-conditioned room of 22 ± 1 °C and 12 h of light. The rats were allowed free access to powdered feed, and mains water that was supplied through an automatic watering system. When they reached 250–300 g, the rats were used for experiments. Rats were divided randomly into groups according to the body weight in a proper range one day before administration of the test substance. All experimental procedures were performed according to the NIH Guide for the Care and Use of Laboratory Animals. The experimental protocol was approved by the Committee on Animal Research, China Medical University.

### 3.2. CCl_4_-Induced Liver Apoptosis

Apoptosis was induced in five groups of 10 rats by an oral administration of 0.5 mL/rat CCl_4_, diluted 1:5 (v/v) in olive oil, twice a week for 8 weeks. The animals received only CCl_4_, CCl_4_ with ESC (10, 100 and 500 mg/kg BW, po, daily) or silymarin (Sigma-Aldrich, Steinheim, Germany; 200 mg/kg BW, daily). The ESC and silymarin were given when the CCl_4_-induced chronic injury model started, and the total drug treatment duration was 8 weeks.

After the blood was drawn from rats at the eighth week, the animals were sacrificed at the same time and the livers were quickly taken out. They were then weighed after being clearly washed with cold normal saline and the moisture sucked up. The largest lobe of liver was divided into two parts for each liver sample, one part was submerged in 10% neutral formalin for the preparation of pathological section, and the other part was stored as a reserve at −80 °C.

### 3.3. Assessment of Liver Functions

The blood was centrifuged at 3024 g (BACKMAN, Krefeld, Germany) at 4 °C for 15 min to separate serum. The levels of serum Alanine Aminotransferase (sALT) and serum Aspartate Aminotransferase (sAST) were assayed using clinical test kits (Roche, Berlin, Germany) spectrophotometrically (Cobas Mira Plus, Frankfurt, Germany).

### 3.4. Antioxidant Enzymes Activity Measurements

Livers were homogenized in nine volumes of isotonic phosphate buffer (0.01 M, pH 7.0). The prepared liver homogenate was centrifuged at 700 g for 5 min at 4 °C. Catalase was assayed by measuring the destruction of H_2_O_2_ at 240 nm according to the method of Aebi [36]. One unit (U) of this enzyme activity is defined as the amount of the enzyme giving K^−1^; where K is the rate constant of the enzyme. Activity is expressed as U per mg of protein (U/mg protein). Superoxide dismutase (SOD) activity was determined according to the method of Misra and Fridovich [37] at room temperature. One hundred microliters of tissue extract was added to 880 μL (pH 10.2, 0.1 mM EDTA) carbonate buffer. Twenty microliters of 30 mM epinephrine (in 0.05% acetic acid) was added to the mixture and measured at 480 nm for 4 min on a Jassco V-550 Spectrophotometer. The enzyme activity was expressed as the amount of enzyme that inhibits the oxidation of epinephrine by 50%, which is equal to 1 unit.

Glutathione peroxidase (GPx) activity was determined according to the method of Flohe and Gunzler [38] at 37 °C. A reaction mixture was composed of 500 μL phosphate buffer, 100 μL 0.01 M GSH (reduced form), 100 μL 1.5 mM NADPH and 100 μL GRx (0.24 units). One hundred microliters of the tissue extract was added to the reaction mixture and incubated at 37 °C for 10 min. Then 50 μL of 12 mM t-butyl hydroperoxide was added to 450 μL tissue reaction mixture and measured at 340 nm for 180 s. The molar extinction coefficient of 6.22 × 10^−3^ M^−1^ cm^−1^ was used to determine the enzyme activity. One unit of activity is equal to the mM of NADPH oxidized/min per mg protein.

### 3.5. Pathological Examinations

For histopathological examination, the formalin fixed liver was embedded in paraffin, cut into 4–5 mm thick sections, stained with hematoxylin-eosin, and observed under a photomicroscope.

### 3.6. Assessment of Liver Apoptosis Proteins and Anti-Apoptic Proteins

Liver tissue was homogenated in PBS buffer at a ratio of 100 mg tissue/0.5 mL PBS for 5 min. The homogenates were placed on ice for 10 min and then centrifuged at 12,000 g for 30 min. Samples containing equal proteins (40 g) were loaded and analyzed by Western blot analysis. Briefly, proteins were separated by 12% SDS-PAGE and transferred onto PVDF membranes (Millipore, Belford, NJ, USA). Membranes were blocked with blocking buffer (5% non-fat dry milk, 20 mM Tris-HCl, pH 7.6, 150 mM NaCl, and 0.1% Tween 20) for at least 1 h at room temperature and then incubated with primary antibodies in the above solution on an orbit shaker at 4 °C overnight. Following primary antibody incubations, membranes were incubated with horseradish peroxidase-linked secondary antibodies (anti-rabbit, anti-mouse, or anti-goat IgG).

### 3.7. Statistical Analysis

Data were expressed as mean ± SEM. Statistical evaluation was carried out by one-way analysis of variance (One-way ANOVA) followed by Scheffe’s multiple range tests. *P* values of less than 0.05 were considered significantly.

## 4. Conclusions

The present study demonstrated that CCl_4_ induced a marked rise in oxidative stress. Our data suggested that mitochondria-initiated apoptosis triggered by ROS plays an important role in CCl_4_-induced hepatotoxicity rats. ESC significantly reduced CCl_4_-induced hepatic apoptosis in rats, probably by exerting a protective effect against hepatocellular apoptosis with its free-radical scavenging ability and inhibiting the Mitochondrial-Dependent Apoptotic Pathway in CCl_4_-induced liver apoptosis in rats.

## Figures and Tables

**Table 1:** Effect of Esculetin and Silymarin on liver catalase, superoxide dismutase (SOD) and glutathione peroxidase (GPx) activities in CCl_4_-treated rats.

Groups	Activity (U/mg protein)		
	Catalase	SOD	GPx
Control	55.82 ± 0.3184	47.26 ± 0.77	64.12 ± 0.72
CCl_4_	28.9 ± 0.484###	24.84 ± 0.36###	24.94 ± 0.46###
CCl_4_ + Silymarin200 mg/kg	33.74 ± 0.47***	31.38 ± 0.71***	30.42 ± 0.81***
CCl_4_ + Esculetin 10 mg/kg	37.76 ± 0.79*	27.4 ± 0.46	28.1 ± 0.55
Cl_4_ + Esculetin 100 mg/g	38.13 ± 1.65*	32.62 ± 0.724***	36.06 ± 0.73***
CCl_4_ + Esculetin 500 mg/kg	41.16 ± 0.64***	32.8 ± 0.55***	40.18 ± 0.44***

Data are expressed as mean ± SEM (n = 10).

* *p* < 0.05,

*** *p* < 0.001 as compared with the CCl_4_ group;

### *p* < 0.001 as compared with the Control group.
